# Teacher violence from the perspectives of teachers and students and related factors: A survey in Southern Iran

**DOI:** 10.3389/fpsyg.2022.942284

**Published:** 2022-11-21

**Authors:** Zahra Foghara Ardestani, Maryam Saber, Mahlagha Dehghan, Abedin Iranpour, Hamideh Baniasadi

**Affiliations:** ^1^Department of Health Education and Promotion, School of Health, Kerman University of Medical Sciences, Kerman, Iran; ^2^Department of Critical Care Nursing, Razi Faculty of Nursing and Midwifery, Kerman University of Medical Sciences, Kerman, Iran; ^3^HIV/STI Surveillance Research Center, WHO Collaborating Center for HIV Surveillance, Institute for Futures Studies in Health, Kerman University of Medical Sciences, Kerman, Iran; ^4^Nursing Research Center, Kerman University of Medical Sciences, Kerman, Iran

**Keywords:** violence, teacher, student, education, schools

## Abstract

**Introduction:**

Teacher violence is defined as the intentional use of power by teachers against students in a variety of ways, including physical, verbal, psychological, and sexual assault. Victims of school violence are more anxious and insecure than other students. An in-depth and comprehensive study on the prevention of violence in schools is urgent and necessary. We conducted this study to determine teacher violence from the perspectives of schoolteachers and students and related factors.

**Methods:**

We conducted this descriptive-analytical study on 313 teachers and 400 students in Ardestan, southern Iran, in 2019. We selected teachers and students using a census method and stratified random sampling, respectively. The research instrument was the school violence questionnaire.

**Results:**

From the perspective of teachers, the mean score of teacher violence (5.7) was significantly lower than that of students (18.3). Male, young, single teachers with diploma and less work experience had significantly higher violence scores than other teachers (*P* < 0.001). We witnessed this trend in public boys’ schools as well (*p* < 0.001). The total mean score for teacher violence was not significantly different between male and female students. From the perspective of students, the mean score of teacher violence was significantly different (*P* < 0.001).

**Conclusion:**

Our results suggest that education authorities and school principals should supervise public school teachers with less education, younger students, and boys’ schools and provide practical training to improve the communication and emotional skills among these teachers.

## Introduction

Nowadays, school violence affects all members of the school community (Nabavi et al., 2017). According to WHO definition, violence is related to any physical, psychosocial and emotional pressure to anybody that lead to injury, hurt and any emotional problem (Yarigholi et al., 2018). Individuals under the age of 18 can develop violence in school (Pajuhi and Nadi, 2017). School violence comes from many sources, so you need to know a lot about them (Devries et al., 2021). On the other hand, many theories such as Bronfenbrenner’s theory believe that relationships are bidirectional, thus violence affect both teachers and students (Navarro and Tudge, 2022). Insults, fight, research on physical and psychological characteristics, socio-economic status, and religious or ethnic affiliation are all examples of violent incidents at school (Cascardi et al., 2018). School violence makes classrooms and school environments unhealthy, makes it hard for teachers to teach, and disrupts the relationship between students and teachers (Yang et al., 2021).

Different levels of school violence are available and teachers frequently use physical and emotional violence against children (McMahon et al., 2017). School violence sometimes associates with teacher’s disengagement, turnover, or emotional wellbeing. In many countries, it is not clear how school violence affects teachers’ profession (Mahdian et al., 2017). Verbal, physical, and psychosocial abuse are all forms of violence (Kord, 2018). many reasons such as teachers’ relation with other staffs, economical and emotional condition of the teachers, job satisfaction and etc. are lead to teacher violence against students, even student behavior against teacher lead to violence too (Ghaderzadeh and Ghaderi, 2016). Studies conducted on physical violence indicated that people, who were angry a lot and did violent behaviors, put a lot of pressure on their bodies, leading to prolonged problems in heart and the nervous system (Payne and Gottfredson, 2019). According to previous studies, aggressive students with no academic achievement provoke their teachers to act violently against them (Choi, 2021). Violence is mostly verbal and occurs frequently in schools with ethnic minorities from marginalized areas (López García et al., 2022). The middle and high schools around the world are full of students who have been the victims of violence from their peers, teachers or other school staff (Jiménez et al., 2021).

Negative consequences of violence include academic difficulty, low self-esteem, school avoidance, depression, and anxiety (Lester et al., 2017). An in-depth and comprehensive study on the prevention of violence in schools is both urgent and necessary (Koposov et al., 2021). Victims of school violence are more anxious and insecure than their peers, and their reaction is to cry and isolate themselves (Wijayaratne, 2020). Victims have low self-esteem and feel pessimistic, humiliated, ashamed, isolated, rejected and unattractive in front of their peers (Vaezi, 2018; Pandey et al., 2021). They have poor social skills and difficulties in their interpersonal relationships, such as social anxiety, loneliness, and fear of being judged negatively (Scharpf et al., 2021). Teachers play an important supportive role in preventing violence in schools, such as assisting students in their lessons (Ferrara et al., 2019). Schools are important places for the development of future students, so we conducted a study to determine teacher violence from the perspectives of teachers and students in elementary and secondary schools and related factors.

## Materials and methods

### Study design and setting

This descriptive-analytical study was performed on 313 teachers and 400 students in Ardestan, southern Iran, in 2020.

### Sample size and sampling

The current study included both teachers and students. A census method was used to sample teachers with at least two years of work experience, with no emotional or psychological problems (self-administered). We invited 313 eligible teachers to participate in the study.

This study included students from elementary and secondary schools. Morgan table was used to select the sample size, so the students’ sample size was 331, but 400 students were considered based on the dropout probability. Stratified random sampling was used to select students. First, a list of schools in Ardestan city (including 84 schools) was prepared and then schools were randomly selected. Nine urban schools were selected for student sampling: two girls’ elementary schools, two boys’ elementary schools, two girls’ junior high schools, one boys’ junior high school, one girls’ high school, and one boys’ high school. We selected teachers from 20 schools, including two boys’ high schools, three boys’ elementary schools, three girls’ elementary schools, and three girls’ junior high schools.

### Measurements

Study tools included a demographic characteristics form and the school violence questionnaire.

Demographic characteristics form consists of age, sex, marital status, level of education, school type, employment type, work experience, etc.

The School Violence Questionnaire assesses violent behavior among students and teachers. This questionnaire shows the teacher’s violent behavior toward students. This scale consists of 36 items based on a 6-point Likert scale (zero = never to five = almost daily). The scores range from 0 to 144, with higher scores reflecting teachers’ high level of violence against students. According to Pişkin et al. (2014), Cronbach’s alpha for the whole scale was 0.96, confirming the reliability of the questionnaire (Cascardi et al., 2018). We confirmed the questionnaire reliability in this study using the Cronbach alpha coefficient of 0.85.

### Data collection

To conduct this research, we visited the research setting and obtained the necessary permissions from the Ardestan Department of Education. We presented the letter of introduction of education to the principals of each school. To collect data, we first explained the study’s objectives and methodology in detail, and then distributed questionnaires to students and teachers in the classroom at the same time. All participants completed demographic characteristics questionnaire at the beginning of the study. To ensure the confidentiality of information and the accuracy of the students’ responses, they completed questionnaires in front of teachers in 25 min.

### Data analysis

Data was analyzed using SPSS22. According to statistician view, descriptive statistics were used to determine mean, standard deviation, frequency, and percent of categorical variables. According to Kolmogorov-Smirnov test, teacher violence scores were not distributed normally. Therefore, the Mann-Whitney U test and Kruskal-Wallis test were used to check the differences in teacher violence between students and teachers and to check the teacher violence scores according to demographic variables. Significance level was considered 0.05.

### Ethical consideration

We conducted this study after receiving an approval from the research department of Kerman University of Medical Sciences and the code of ethics No. IR.KMU.REC.1398.459. All participants completed informed consent to participate in the study. We assured teachers and students to keep their information confidential.

## Results

Teachers had an average age of 36.7 years, ranging from 23 to 56 years, and an average work experience of 15.69 years. The majority of teachers in the present study were female (67.4%) and married. Seventy-three point five percent of the teachers participating in the study had a bachelor’s degree (Table 1).

**TABLE 1:** The relationship between characteristics of teachers and teacher violence.

Variable		Frequency (%)	Teacher violence		Statistical test	*P*-value
				
			Mean	SD		
Gender	Female	211 (67.4)	4.51	8.8	Z = −4.63	< 0.001
	Male	102 (32.6)	8.33	11.05		
Marital status	Single	36 (11.5)	7.5	7.82	Z = −3.18	0.001
	Married	277 (88.5)	5.53	9.96		
Level of education	Diploma	4 (1.3)	10.0	3.46	H = 12.68	0.005
	Associate degree	26 (8.3)	6.35	13.23		
	Bachelor	230 (73.5)	5.23	9.2		
	Above bachelor	53 (16.9)	7.45	10.32		
Spouse’s education level	Diploma	60 (21.7)	3.82	7.16	H = 3.39	0.34
	Associate degree	45 (16.3)	4.18	5.89		
	Bachelor	150 (54.3)	6.29	11.11		
	Above bachelor	21 (7.7)	7.71	14.04		
Spouse occupation	Education staff	57 (20.7)	6.96	10.67	H = 5.67	0.34
	Worker	8 (2.9)	3.25	4.92		
	Self-employed	82 (29.7)	4.27	9.44		
	Clerk	76 (27.5)	5.29	9.61		
	Retired	12 (4.3)	4.75	9.65		
	Unemployed	41 (14.9)	7.1	11.42		
Grade	Elementary school	135 (43.1)	5.44	10.04	H = 2.63	0.27
	Junior high school	88 (28.1)	5.94	9.96		
	High school	90 (28.8)	6.04	9.14		
Type of school	Girls’ state school	172 (55.0)	4.98	10.36	H = 18.61	< 0.001
	Girls’ private school	15 (4.8)	7.4	14.29		
	Boys’ state school	120 (38.3)	6.79	8.15		
	Boys’ private school	5 (1.6)	2.6	4.77		
Type of employment	Hired	257 (82.1)	5.75	9.79	H = 0.07	0.97
	Contract recruiter	11 (3.5)	6.82	10.39		
	Tuition	45 (14.4)	5.51	9.53		
Financial satisfaction	Yes	95 (30.4)	4.28	7.19	H = 5.61	0.06
	Partly	105 (33.5)	6.52	10.68		
	No	113 (36.1)	6.28	10.6		
School location	Downtown	127 (40.5)	6.27	9.62	H = 3.11	0.21
	Uptown	147 (47.0)	5.71	10.62		
	Suburbs	39 (12.5)	4.26	6.04		

SD: Standard deviation; Z = Mann-Whitney U test, H = Kruskal-Wallis test.

The mean age of students was 13.8 ± 2.42, ranging from 10 to 18 years. Fifty-nine percent of the students in the study were girls. Thirty-three point eight percent of the students were in elementary school, 38.4% were in junior high school, and 27.8% were in high school (Table 2).

**TABLE 2:** The relationship between characteristics of students and teacher violence.

Variable		Frequency (%)	Teacher violence		Statistical test	*P*-value
				
			Mean	SD		
Sex	Girl	236(59)	17.33	19.12	Z = -1.52	0.13
	Boy	164(41)	19.7	19.16		
Grade	Elementary school	135(33.8)	14.69	17.02	H = 17.33	<0.001
	Junior high school	154(38.4)	22.16	20.62		
	High school	111(27.8)	17.33	18.36		

SD: Standard deviation; Z = Mann-Whitney U test, H = Kruskal-Wallis test.

The mean scores of teacher violence from the perspectives of teachers and students were 5.76 ± 9.74 and 18.30 ± 19.15, respectively, with a significant difference between teachers and students in this regard (Table 3). Among all the items of the Teacher Violence Questionnaire, the items of “ear twisting” and “standing on one foot in the classroom” were not significantly different from the perspectives of teachers and students. The other items received higher ratings from the students’ perspective than the teachers. From the perspective of teachers, teacher violence items scores ranged from 0.03 to 0.54. The most violent behavior, according to teachers, was “threatening to give low grades or fail students.” From the perspective of students, the teacher violence items scores ranged from 0.19 to 0.9. The most violent behavior, according to students, was “ignoring hand raisers or not answering students’ questions.” According to the expected range of the Teacher Violence Questionnaire, which was between 0 and 144, we found that teacher violence from the perspectives of teachers and students was very low (Table 3).

**TABLE 3:** Comparison of teacher violence from the perspectives of teachers and students.

Group	Teacher violence				Mann-Whitney test	*P*-value
			
items	Teachers		Students			
				
	Mean	SD	Mean	SD		
(1). Pulling hair	0.15	0.48	0.53	0.96	−5.92	< 0.001
(2). Ear twisting	0.17	0.47	0.2	0.61	−0.2	0.84
(3). Slapping in the face	0.18	0.52	0.6	1.0	−6.47	< 0.001
(4). Punching	0.18	0.54	0.6	1.05	−6.22	< 0.001
(5). Tapping the head	0.14	0.5	0.42	0.89	−5.33	< 0.001
(6). Smashing the heads of two students	0.05	0.23	0.2	0.65	−3.09	0.002
(7). Hitting the student’s head against the wall or table	0.07	0.31	0.19	0.59	−2.92	0.004
(8). Kicking	0.21	0.62	0.76	1.11	−8.0	< 0.001
(9). Beating a student with a tool such as a stick or ruler, etc.	0.18	0.53	0.42	0.92	−3.58	< 0.001
(10). Throwing some objects at the student	0.14	0.5	0.64	1.04	−8.15	< 0.001
(11). Forcing students to stand on one foot in class	0.17	0.5	0.25	0.68	−1.22	0.22
(12). Mocking a student with physical characteristics (height, weight, teeth, skin color, etc.)	0.12	0.44	0.84	1/2	−10.34	< 0.001
(13). Mocking a student with a personal appearance (clothes, glasses, etc.)	0.1	0.36	0.64	1.07	−8.49	< 0.001
(14). Mocking the student’s accent, dialect, pronunciation style	0.13	0.41	0.66	1.09	−7.94	< 0.001
(15). Mocking a first or last name	0.08	0.34	0.66	1.14	−8.64	< 0.001
(16). Offensively calling a student name or nickname	0.13	0.43	0.69	1.11	−8.62	< 0.001
(17). Blaming the whole class or group you are in.	0.28	0.65	0.66	1.09	−4.91	< 0.001
(18). Calling students with rude words (stupid, etc.)	0.18	0.53	0.82	1.17	−8.68	< 0.001
(19). Constantly searching for faults	0.13	0.43	0.51	0.98	−5.86	< 0.001
(20). Accusing a student for no reason	0.13	0.43	0.43	0.9	−5.45	< 0.001
(21). Having a scornful look at the student	0.2	0.57	0.46	0.87	−4.32	< 0.001
(22). Threatening to give low grades or fail students	0.54	0.91	0.82	1.28	−1.99	0.047
(23). Humiliating students in front of their classmates (mocking homework or exam paper.)	0.26	0.6	0.59	1.01	−4.42	< 0.001
(24). Ignoring hand raisers and not answering a student’s question	0.32	0.66	0.9	1.22	−6.89	< 0.001
(25). Giving additional duties as punishment	0.44	0.88	0.72	1.3	−3.56	< 0.001
(26). Restricting student’s freedom (teacher does not allow the student to go outside the classroom during the break)	0.23	0.58	0.75	1.11	−7.47	< 0.001
(27). Creating a negative mindset in students about another student.	0.15	0.47	0.54	1.0	−6.27	< 0.001
(28). Complaining about a student to the school principal unfairly	0.13	0.43	0.58	1.03	−7.23	< 0.001
(29). Disclosing personal and private information	0.17	0.5	0.35	0.84	-2.93	0.003
(30). Tearing personal belongings (books, notebooks or paintings.)	0.13	0.44	0.34	0.8	−3.9	< 0.001
(31). Making sex jokes with students	0.05	0.34	0.31	0.87	−5.45	< 0.001
(32). Getting the student to talk about sex	0.03	0.28	0.20	0.7	−4.55	< 0.001
(33). Calling students with sexual words	0.04	0.34	0.2	0.66	−4.82	< 0.001
(34). Making sexual cues with hand, arm, and eye movements.	0.04	0.33	0.27	0.73	−5.89	< 0.001
(35). Touching students inappropriately	0.04	0.26	0.2	0.66	−4.84	< 0.001
(36). Creating and promoting immoral rumors among students	0.04	0.27	0.32	0.82	−6.95	< 0.001
Total score	5.76	9.74	18.3	19.15	−11.39	< 0.001

SD: Standard deviation.

We found a significantly poor correlation between teacher violence, age (Spearman correlation coefficient = −0.13, *p*-value = 0.017), and work experience of teachers (Spearman correlation coefficient = −0.13, *p*-value = 0.02). The score of teacher violence was significantly different in terms of gender, marital status, level of education, and type of school (*P* < 0.001). Men had a higher mean score for teacher violence than women, and singles had a higher score than married people (*P*-value = 0.001) (*P*-value = 0.001). Teachers with a diploma had a higher rate of teacher violence than other teachers (*P*-value = 0.005) (*P*-value = 0.005). Teachers in boys’ state schools had higher levels of violence than teachers in other schools (*P*-value < 0.001).

We indicated a significantly direct and poor correlation between students’ age and teacher violence (Spearman correlation coefficient = 0.12 and *P* value 0.018). As students grew older, so did their views on the prevalence of teacher violence, and vice versa. The mean score of teacher violence was not significantly different from the perspectives of male and female students. The mean score of teacher violence was significantly different from the perspective of students at different levels (*P* < 0.001). The score of teacher violence from the perspective of junior high school students was significantly higher than that of other high school students (Table 2).

## Discussion

Our results suggested that from the perspective of teachers, the mean score of teacher violence was significantly lower than that from students’ perspective. Vaezi (2018) indicated that students’ experiences of violence in the education system took the form of “harmful education system,” “school dropout,” and “application of care strategies.” They emphasized the importance of preventive measures against violence at different levels in the education system. Enactment of violence against persons act in the educational system, rehabilitation of injured children, reduction and control of violence in the educational system are effective and preventive measures (Vaezi, 2018). Teachers considered lower average violence than students because they reported less violence and believed that students punishment was so useful for them to act better (Cluver et al., 2018), even those who experienced violence behaved more aggressively (Scharpf et al., 2021). Suryaningrat et al. (2020) found that aggressive behavior had a direct relationship with aging (Suryaningrat et al., 2020).

We revealed that from the perspective of students, the most violent behavior was “ignoring hand raisers or not answering students’ questions,” while from the perspective of teachers, the most violent behavior was “threatening students to give them lower grades or fail them.” Pajuhi indicated that from the perspective of the students, “blaming the whole class or the group that you are in” had the highest mean score of teacher violence. Rerkswattavorn and Chanprasertpinyo (2019) reported that many teachers tended to do verbal violence than physical violence (Rerkswattavorn and Chanprasertpinyo, 2019).

Our results suggested that men had a higher mean score of teacher violence than women and single people had higher scores than married people. Male teachers seem to have less self-control and violence control because they are less sociable and friendly (Pajuhi and Nadi, 2017). On the other hand, males are more use of aggressive behaviors than females, maybe this difference origin in culture that people expect men that they are tough and inflexible and women are more emotional and they are not nurtured to be harsh (Yarigholi et al., 2018). Also, teachers who are single, perhaps they do not complete their socialization process and they do not manage their behaviors. In addition to, married teacher maybe have children, thus they act compassionately and their patience are more than singles, because they learned formerly (Dehghan, 2016). Previous studies mentioned that emotional condition, teacher well-being, and stress level of teachers caused them to behave aggressively (Miles et al., 2016; Hecker et al., 2018). Working condition is one source of acting violently against students (Scharpf et al., 2021).

Teachers with diploma had higher violence than other teachers did. It should to mention that level of education of teacher is effect on violence, this result is originated that in university many course that belong to psychosocial problems and they learn how they can control their feelings or when they were placed with this situation how they can do the best, so teachers that have diploma and do not have academic education are more susceptible to use violence (Tuna and Aslan, 2018). Ceballos and Carvalho (2019) indicated that low physical work related to physical and verbal violence, theft and robbery, and low emotional ability had a relationship with physical and verbal violence, usage of a weapon, and some types of violence. We found a correlation between the physical, emotional, and future work ability of teachers and school violence, indicating the need to promote a safer environment for work inside the school and in society as a whole (Ceballos and Carvalho, 2019). Romero et al. (2018) demonstrated that teachers’ academic support from adolescents was low in poorly resourced schools. Secondary prevention programs in schools provide students with additional training and academic support in disadvantaged areas, so they can reduce violence and the socioeconomic consequences of adolescents’ school delay (Romero et al., 2018). Fabbri et al. (2021) demonstrated that teachers with low salary acted more aggressively (Fabbri et al., 2021). Devries et al. (2021) believed that economical condition, availability of facilities, a large number of students and supporting system for teaching affected teachers’ behaviors (Devries et al., 2021). Yang et al. (2021) indicated the significant and negative impacts of school violence on teacher professional engagement mediated by teacher self-efficacy. We can alleviate school violence by enhancing participation of school stakeholders and improving teacher–student relationships (Yang et al., 2021).

Public boys’ schools had higher violence scores than other schools. Shirbegi and Moradi (2017) found that the intensity of inappropriate interactions and coercion between male principals was different from female principals, so male principals were more violent and sometimes used illegal power to solve problems. Studies showed that boys’ different physical appearance, societal tolerance, and biological differences might explain some differences in levels of violence between males and females (Butchart et al., 2015; Golshiri et al., 2018). The type of communication between the education system and learners in public schools (vertical and top-down communication) may lead to perception of a higher level of violence in students. Kapa et al. (2018) reported that school personnel should enforce school rules and reduce negative issues in each school, such as student truancy and apathy. As high levels of support reduce instances of violence, these findings have important implications for school environments.

## Conclusion

Our results indicated that teachers and students had different perspectives on violence. Teachers reported lower violence than students did because they were unaware that their behavior was a form of violence against students or they concealed their violence. In line with this finding, it should necessary that demographic characteristics of teachers like gender, marital status, level of education and etc. are considered and assess related factors more. Violence against children is a significant cause of physical and psychological problems. Governments should guide teachers how to communicate with students properly. Governments should enact a bill to protect children. The adoption of the most effective teaching methods across the educational system and support of teachers to improve non-violent and positive strategies could be the best ways to protect children from all forms of violence in all settings, including schools.

## Limitation

Fatigue and boredom of teachers in the last hours of the school time is one of the most common limitations in current study; therefore, to overcome this problem, we tried to attend before start of classes. Another limitation of this study was that some teachers did not care about us, thus we talked to them until they agreed to cooperate. All data were self-reported by teachers, so their self-enhancement biases might have affected the objectivity of the responses. Cultural traits, variation in school and educational management or other characteristics associated with the variance of teacher professional engagement may all be significant. Thus, any cause and effect implication remains unclear. On the other hand, this study is cross-sectional study that many factors may be neglected. Therefore, it is necessary to advance in longitudinal studies that allow for greater explanatory power.

## Data availability statement

The raw data supporting the conclusions of this article will be made available by the authors, without undue reservation.

## Ethics statement

The studies involving human participants were reviewed and approved by Kerman University of Medical Sciences. Written informed consent to participate in this study was provided by the participants’ legal guardian/next of kin.

## Author contributions

MS, MD, and AI: conceptualization, supervision, methodology, data analysis, and writing—reviewing and editing. ZA and HB: conceptualization, data curation, software, and writing—original draft preparation. All authors contributed to the article and approved the submitted version.
